# Co-Exposure to SiO_2_ Nanoparticles and Arsenic Induced Augmentation of Oxidative Stress and Mitochondria-Dependent Apoptosis in Human Cells

**DOI:** 10.3390/ijerph16173199

**Published:** 2019-09-01

**Authors:** Maqusood Ahamed, Mohd Javed Akhtar, Hisham A. Alhadlaq

**Affiliations:** 1 King Abdullah Institute for Nanotechnology, King Saud University, Riyadh 11142, Saudi Arabia; 2 Department of Physics and Astronomy, College of Science, King Saud University, Riyadh 11142, Saudi Arabia

**Keywords:** combined toxicity, SiO_2_ nanoparticles, arsenic, human health, oxidative stress, apoptosis

## Abstract

Widespread application of silica nanoparticles (nSiO_2_) and ubiquitous metalloid arsenic (As) may increase their chances of co-exposure to human beings in daily life. Nonetheless, studies on combined effects of nSiO_2_ and As in human cells are lacking. We investigated the co-exposure effects of nSiO_2_ and As in human liver (HepG2) and human fibroblast (HT1080) cells. Results showed that nSiO_2_ did not cause cytotoxicity. However, exposure of As caused oxidative stress and apoptosis in both types of cells. Interesting results were that co-exposure of a non-cytotoxic concentration of nSiO_2_ significantly augmented the As induced toxicity in both cells. Intracellular level of As was higher in the co-exposure group (nSiO_2_ + As) than the As group alone, suggesting that nSiO_2_ facilitates the cellular uptake of As. Co-exposure of nSiO_2_ and As potentiated oxidative stress indicated by pro-oxidants generation (reactive oxygen species, hydrogen peroxide and lipid peroxidation) and antioxidants depletion (glutathione level, and glutathione reductase, superoxide dismutase and catalase activities). In addition, co-exposure of nSiO_2_ and As also potentiated mitochondria-mediated apoptosis suggested by increased expression of *p53*, *bax*, *caspase-3* and *caspase-9* genes (pro-apoptotic) and decreased expression of *bcl-2* gene (anti-apoptotic) along with depleted mitochondrial membrane potential. To the best of our knowledge, this is the first study showing that co-exposure of nSiO_2_ and As induced augmentation of oxidative stress and mitochondria-mediated apoptosis in HepG2 and HT1080 cells. Hence, careful attention is required for human health assessment following combined exposure to nSiO_2_ and As.

## 1. Introduction

Nano-scale silica (nSiO_2_) is one of the most widely used nanoparticles due to its extraordinary properties such as monodispersity, drug loading capacity and potential to hybridize with other organic or inorganic materials [1,2]. These unique properties of nSiO_2_ offer great potential for various applications e.g., cosmetics, food industry, environmental remediation, drug delivery, biosensor and tissue imaging [3,4,5]. Due to high production and broad applications release of nSiO_2_ to the natural environment and potential risk of their toxicity is inevitable. Biodistribution and toxicity of nSiO_2_ have been extensively studied. In general, nSiO_2_ is not toxic at low exposure level but exerts toxicity at high exposure level [6,7,8]. Earlier reports demonstrate that nSiO_2_ induces inflammation, oxidative stress and cell death to different mammalian cells [9,10,11]. The nSiO_2_ is also able to translocate into the blood stream and cause toxicity to various vital organs including lung, liver, heart and brain [12,13].

Metalloid arsenic (As) is a ubiquitous environmental contaminant, whose risk of human poisoning is a global concern [14]. The Agency for Toxic Substances and Diseases Registry (ATSDR) ranks As first in the priority list of hazardous substances [15]. Recent statistics show that global production of As in 2018 was around 37,000 ton/year, hence, increased As disposal to the environment [16]. Inorganic As is more toxic than organic As. Among inorganic, As (III) is more toxic than As (V) [17]. However, toxicity of As might largely be affected by several environmental factors including organic matters, colloids and presence of nanoparticles [18,19]. Therefore, these factors should be taken account while evaluating the toxicity of As [20,21].

Current research is now focusing on interaction of nanoparticles with pre-existing environmental contaminants, which may further enhance the undesirable effects to human health. Hence, studies on interaction of nSiO_2_ with pre-existing contaminants have a practical importance in the field of toxicology [22]. Most of the toxicological studies on nSiO_2_ are focused on single exposure and research on combined effects of nSiO_2_ and pre-existing environmental pollutants are limited. Wu et al. [23] observed that co-exposure of nSiO_2_ and benzo(a)pyrene causes synergistic toxicity to human bronchial epithelial (BEAS-2B) cells. Co-exposure of nSiO_2_ and methylmercury (MeHg) or lead (Pb) induces synergistic cardiac toxicity [24,25]. The nSiO_2_ and Pb co-exposure induces joint toxicity to human lung epithelial cells (A549) [26,27].

Extensive applications of nSiO_2_ and ubiquitous As contamination may increase their chance of co-exposure to humans in daily life. For instance, As contaminated drinking water significantly contributes to total As intake by the general population of many countries [28]. These populations may also consume nSiO_2_ polluted water or food. Therefore, it is imperative to explore the co-exposure effects of nSiO_2_ and As in human cells. Studies on combined effects of nSiO_2_ and As in human cells are lacking. Hence, we investigated the co-exposure effects of nSiO_2_ and As in human liver HepG2 and human fibroblast HT1080 cells. Liver is one of the target organs for As and nSiO_2_ [29,30]. Xie et al. [30] also demonstrated that nSiO_2_ mainly accumulates in liver, lung and spleen after intravenous administration. We have also chosen the HT1080 cell line to evade cell type specific responses following co-exposure of nSiO_2_ and As. The HepG2 and HT1080 cell lines have been widely used in toxicity studies [31,32,33]. To achieve this goal, we examined the various biomarkers of cytotoxicity, oxidative stress and apoptosis in both HepG2 and HT1080 following exposure to nSiO_2_ and/or As. The underlying mechanisms of combined toxicity of nSiO_2_ and As were also discussed.

## 2. Materials and Methods

### 2.1. SiO_2_ Nanoparticles and Arsenic Preparation

Amorphous SiO_2_ nanopowder (size: 10–20 nm, purity: 99.5% trace metals basis) and sodium arsenate (Na_2_HAsO_4_·7H_2_O) (As (V)) were purchased from Sigma-Aldrich (St. Louis, MO, USA). A stock solution (1 mg/mL) of nSiO_2_ was prepared in distilled water. In order to avoid the agglomeration, the solution was sonicated using a sonicator at 40 W for 5 min before adding to culture medium. Sodium arsenate was used as a source of As (V) and dissolved in distilled water.

Amorphous nSiO_2_ was characterized in our laboratory before toxicity studies. The amorphous nature of nSiO_2_ was indicated by X-ray diffraction (XRD) (PANalytical X’Pert) assembled with Ni filter and Cu Kα(λ = 1.54056 Å) radiations as a source of X-ray. Field emission scanning electron microscopy (FESEM, JSM-7600F, JEOL, Inc., Japan) and field emission transmission electron microscopy (FETEM, JEM-2100F, JEOL, Inc.) were used to examine the morphology of nSiO_2_. ZetaSizerNano-HT (Malvern Instruments, UK) was applied to measure the hydrodynamic size and zeta potential of nSiO_2_ in distilled water and culture medium.

### 2.2. Cell Culture

The HepG2 and HT1080 cell lines were cultured in Dulbecco’s modified Eagle’s medium (DMEM) (Invitrogen, CA, USA) with 100 unit/mL of antibiotics (penicillin–streptomycin, Invitrogen) and 10% fetal bovine serum (FBS) (Invitrogen) at 37 °C with 5% CO_2_ supplementation. At appropriate confluence (80%–85%), cells were harvested using trypsin (Invitrogen) and sub-cultured for further experiments.

### 2.3. Selection of Appropriate Concentration of nSiO_2_ and As

Screening tests (3-(4, 5-dimethylthiazol-2-yl)-2, 5-diphenyltetrazoliumbromide (MTT) assay) were performed to define the appropriate concentrations of nSiO_2_ and As for the assessment of their combined effects in HepG2 and HT1080 cells. Both types of cells were exposed to various concentrations of nSiO_2_ (0.5, 1, 5, 10, 25, 50 and 100 μg/mL) and As (0.1, 0.2, 0.5, 1, 2, 5 and 10 μg/mL) for 24 h. Results demonstrated that nSiO_2_ did not induce cytotoxicity to both HepG2 and HT1080 cells in selected concentrations. On the other hand, heavy metal As induced concentration-dependent cytotoxicity in both HepG2 and HT1080 cells in the concentration range of 0.1–10 μg/mL. We further examined the co-exposure effects of nSiO_2_ and As with different combinations. Based on these results, we selected one concentration of each material; non-cytotoxic concentration of nSiO_2_ (10 μg/mL) and cytotoxic concentration of As (1 μg/mL) to explore their combined effects in HepG2 and HT1080 cells (screening data not given).

### 2.4. Cytotoxicity Parameters

Cells were seeded in 96-well plate and exposed for 24 h to either nSiO_2_ (10 μg/mL), or As (1 μg/mL) or a combination of both (SiO_2_ + As). Cell viability was examined by 3-(4, 5-dimethylthiazol-2-yl)-2, 5-diphenyltetrazoliumbromide (MTT) and neutral red uptake (NRU) assays with specific medications [34]. In an MTT assay, live cells have ability to reduce MTT into a blue formazan compound. Absorbance of this compound was recorded at 570 nm (Microplate reader, Synergy-HT, Biotek, Vinooski, VT, USA). The NRU assay is based on the ability of live cells to integrate and bind neutral red (NR) dye with lysosomes. The absorbance of NR probe was measured at 540 nm (Microplate reader (Synergy-HT, Biotek)).

### 2.5. Measurement of Intracellular Level of As

Inductively coupled plasma mass spectrometry (ICP-MS) was used to determine the effect of nSiO_2_ on uptake of As in HepG2 and HT1080 cells. Briefly, both types of cells were exposed for 24 h to either nSiO_2_ (10 μg/mL), or As (1 μg/mL) or both (SiO_2_ + As). At the completion of exposure, cells were washed two times with phosphate buffer saline PBS and harvested. After centrifugation cell pellets were digested with nitric acid. Then, the digested solution was dissolved in 4% nitric acid for the measurement of As content using ICP-MS. The intracellular level of As was represented in picogram (pg)/cell.

### 2.6. Interaction of nSiO2 and As in Culture Media

Interaction of nSiO_2_ and As in culture medium (DMEM) was also assessed by ICP-MS. Briefly, three groups of samples were prepared; As group (1 μg/mL), co-exposure group (10 μg/mL of nSiO_2_ and 1 μg/mL of As) and control group (DMEM only). All the samples were incubated for 0 h and 24 h with mild shaking. High speed centrifugation was done to obtain clear supernatants. Supernatants were then digested with nitric acid (HNO_3_) and As content was determined by ICP-MS. Level of As adsorbed on the surface of nSiO_2_ was equal to decreased level of As in supernatant over 0 and 24 h.

### 2.7. Oxidative Stress Parameters

Several markers of oxidative stress were assessed in HepG2 and HT1080 cells exposed for 24 h to either nSiO_2_ (10 μg/mL), or As (1 μg/mL) or both (SiO_2_ + As). Dichlorofluorescin diacetate (DCFH-DA) probe was used to measure the reactive oxygen species (ROS) generation in cells as per the instruction of a previous report [31]. The DCFH-DA probe passively enters the cells and reacts with ROS to form a fluorescent compound named dichlorofluorescein (DCF). Fluorescent intensity of DCF was assessed by two distinct methods; qualitative analysis by fluorescent microscope (DMi8, Leica Microsystems, Germany) and quantitative measurement by a microplate reader (Synergy-HT, Biotek). The intracellular hydrogen peroxide (H_2_O_2_) level was assessed using a commercially available kit (Sigma-Aldrich). Malondialdehyde (MDA, a lipid peroxidation marker) was determined as per the protocol of Ohkawa et al. [35]. The intracellular level of glutathione (GSH) was determined by Ellman’s method [36] using 5, 5-dithio-bis-2-nitrobenzoic acid (DTNB). Glutathione reductase (GR) enzyme activity was assayed by recording the decrease in absorbance of nicotinamide adenine dinucleotide phosphate (NADPH) as reported by [37]. A commercial kit from Cayman Chemical Company (Michigan, OH, USA) was applied to assay the activity of superoxide dismutase (SOD) enzyme. Protocol from Sinha et al. [38] was used to assay the activity of catalase (CAT) enzyme.

### 2.8. Apoptotic Markers

Combined effects of nSiO_2_ and As on apoptotic genes and enzymes, cell cycle and mitochondrial membrane potential (MMP) were assessed in HepG2 and HT1080 cells following 24 h treatment to either nSiO_2_ (10 μg/mL), or As (1 μg/mL) or both (SiO_2_ + As). Real-time PCR (ABI PRISM 7900HT Sequence Detection System, Applied Biosystems, Foster City, CA) was applied to examine the expression of mRNA level of apoptotic genes (*p53*, *bax*, *bcl-2*, *casp3* and *casp9*) using SYBR green. Protocol and the specific set of primers sequence are reported in earlier work [34]. Activity of caspase-3 and caspase-9 enzymes was assessed using BioVision colorimetric kit (Milpitas, CA, USA). The cationic fluorochrome rhodamine-123 (Rh-123) probe was used to assess the MMP level [31]. The Rh-123 binds to the mitochondria of living cells in a membrane potential-dependent fashion. Fluorescent intensity of Rh-123 (MMP level) was determined by two distinct procedures; qualitative assessment using a fluorescent microscope (DMi8, Leica Microsystems, Germany) and quantitative analysis using a microplate reader (Synergy-HT, Biotek). A propidium iodide (PI) fluorescent probe was applied to analyze cell cycle phases using a flow cytometer (Coulter Epics XL/Xl-MCL) via F1-4 filter (585 nm) [31].

### 2.9. Protein Assay

Cellular protein content was assayed by Bradford’s protocol [39].

### 2.10. Statistics

One-way analysis of variance (ANOVA) followed by Dunnette’s multiple comparison tests were applied for the statistical analysis of results. The *p* < 0.05 was attributed as statistical significance. All the analyses were done utilizing the Prism software package (GraphPad Software, Version 5.0, GraphPad Software Inc., San Diego, CA, USA).

## 3. Results and Discussion

### 3.1. Characterization of nSiO_2_

**Figure 1:**
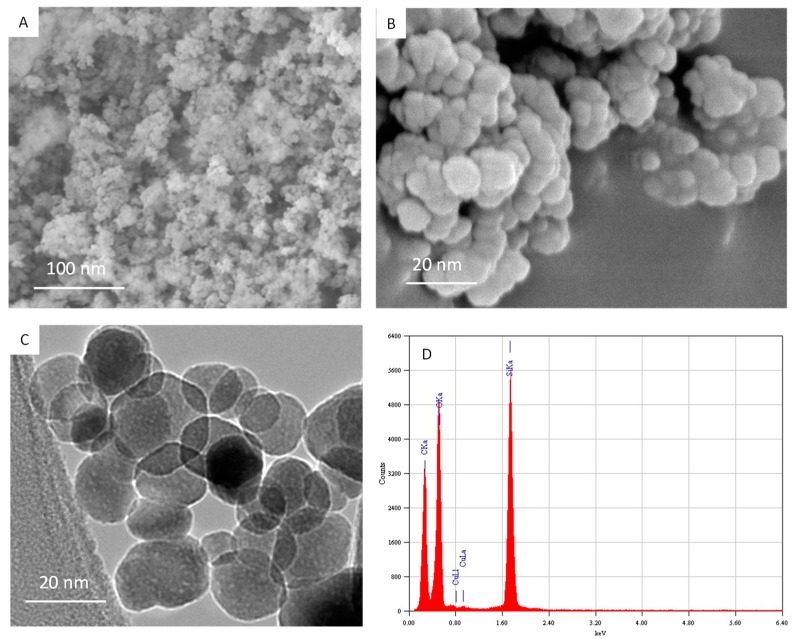
Characterization of nSiO_2_. (**A**,**B**) SEM images of nSiO_2_. (**C**) TEM image of nSiO_2_. (**D**) Elemental composition of nSiO_2_ analyzed by energy dispersive X-ray spectroscopy (EDS).

Characterization of amorphous nSiO_2_ was done by XRD, SEM, TEM and energy dispersive X-ray spectroscopy (EDS) techniques. The amorphous nature of nSiO_2_ was confirmed by XRD (data not shown). SEM images suggested smooth surfaces of nSiO_2_ (Figure 1A,B). Figure 1C represents the TEM image of nSiO_2_. These images suggested that nSiO_2_ were almost spherical shaped. The mean particle size was calculated after measuring over 100 particles from the TEM image. The average particle size of nSiO_2_ was approximately 15 nm. Figure 1D presents the elemental composition of nSiO_2_ analyzed by EDS. The EDS spectra indicated that Si and O were main elemental composition in nSiO_2_. Elemental impurities were not detected. The presence of C and Cu peaks was due to the carbon coated Cu grid of TEM.

Zeta potential and hydrodynamic size of nSiO_2_ in distilled water and DMEM were determined at various time intervals (Table 1). Hydrodynamic sizes were 5–6 times higher than the size calculated from TEM. This might be due to agglomeration of particles in aqueous state [8]. Zeta potential data provide quantitative information on the dispersion and stability of particles in aqueous medium. It is reported that particles show good dispersion and stability when zeta potential values are higher than 30 mV [24,40]. In this study, nSiO_2_ exhibited excellent dispersion in both distilled water and culture medium as zeta potential values were more than 30 mV (Table 1).

### 3.2. Cytotoxicity Study

**Figure 2:**
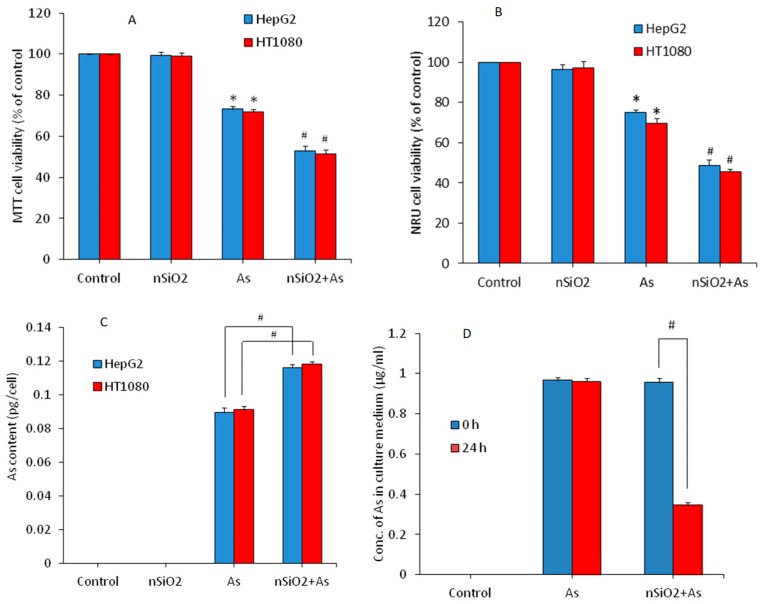
Cytotoxicity of HepG2 and HT1080 cells exposed for 24 h to silica nanoparticles (nSiO_2_) (10 µg/mL), As (1 µg/mL) or SiO_2_ + As (10 µg/mL + 1 µg/mL). (**A**) 3-(4, 5-dimethylthiazol-2-yl)-2, 5-diphenyltetrazoliumbromide (MTT) assay. (**B**) Neutral red uptake (NRU) assay. (**C**) Intracellular level of As in HepG2 and HT1080 cells exposed to nSiO_2_ (10 µg/mL), As (1 µg/mL) or SiO_2_ + As (10 µg/mL + 1 µg/mL) for 24 h. (**D**) Adsorption of As on the surface of nSiO_2_ in culture medium. Data are presented as the mean ± SD of three independent experiments (n = 3). * indicates significant effect in comparison to the control group (*p* < 0.05). ^#^ indicates significant effect in comparison to nSiO_2_ group alone or As group alone *(p* < 0.05).

Combined cytotoxicity of nSiO_2_ and As was examined in HepG2 and HT1080 cells following exposure to either nSiO_2_ (10 μg/mL), or As (1 μg/mL) or a combination of nSiO_2_ + As (10 μg/mL + 1 μg/mL) for 24 h. Figure 2A shows that nSiO_2_ did not induce cytotoxicity, however, As significantly decreased the cell viability of both types of cells (73.2% for HepG2 and 71.8% for HT1080) in comparison to the control group (*p* < 0.05). Interestingly, in co-exposure group (nSiO_2_ + As) cytotoxicity was more pronounced (52.9% for HepG2 and 51.3% for HT1080) as compared to As group alone (*p* < 0.05). NRU data also showed that nSiO_2_ was not toxic, however, As significantly decreased the cell viability in comparison the control group (Figure 2B). Again, in the co-exposure group (nSiO_2_ + As), cell viability reduction was significantly higher than those of the As group alone (*p* < 0.05) (Figure 2B). These results suggested that non-cytotoxic dosage of nSiO_2_ potentiated the cytotoxic response of As in both HepG2 and HT1080 cells. Previous studies also report that non-cytotoxic concentrations of nSiO_2_ enhances cytotoxicity and apoptosis response of lead (Pb) in A549 cells [26,27]. Yang et al. [24] observe that exposure of nSiO_2_ significantly increases the cardiac toxicity of methylmercury (MeHg) in human cardiomyocytes and rat heart tissue. Besides, toxicity of As in *Daphnia magna* and fresh-water algae (*Microcystis aeruginosa* and *Scenedesmus obliquus*) was enhanced by TiO_2_ nanoparticles [20,21].

How a non-cytotoxic concentration of nSiO_2_ potentiated toxicity of As in HepG2 and HT1080 cells in this study was not clear at this point. Hence, we further examined the effect of nSiO_2_ on cellular uptake of As. An earlier report shows that nSiO_2_ internalizes into HepG2 cells through an endocytosis process [41]. Intracellular levels of As were measured by ICP-MS. Both the HepG2 and HT1080 cells were exposed to either nSiO_2_ (10 μg/mL) or As (1 μg/mL) or a combination of both (nSiO_2_ + As) for 24 h. Figure 2C demonstrated that the co-exposure group (nSiO_2_ + As) had higher intracellular As content as compared to the As group alone, suggesting that nSiO_2_ facilitated the cellular uptake of As in both types of cells. To confirm this, we further examined the adsorption efficiency of nSiO_2_ for As in culture medium without cells. Figure 2D demonstrated that most of the As present in culture media was adsorbed on the surface of nSiO_2_ in the co-exposure group (nSiO_2_ + As). This notion might explain the facilitated cellular uptake of toxic metals adsorbed on the surface of nSiO_2_. This could be the possible explanation behind the higher toxicity in the co-exposure group (nSiO_2_ + As) than those of the As group alone. Guo et al. [42] found that combined exposure of a low toxic dose of nSiO_2_ and Cd noticeably increases Cd accumulation in mice liver and augments the hepatotoxicity of Cd. Higher intracellular levels of MeHg in the co-exposure group (nSiO_2_ + MeHg) compared to cells exposed to MeHg alone is also observed [43]. Limbach et al. [44] suggest that nSiO_2_ act as a “Trojan horse” in cells co-exposed to manganese (Mn) or cobalt (Co).

### 3.3. Oxidative Stress Study

**Figure 3:**
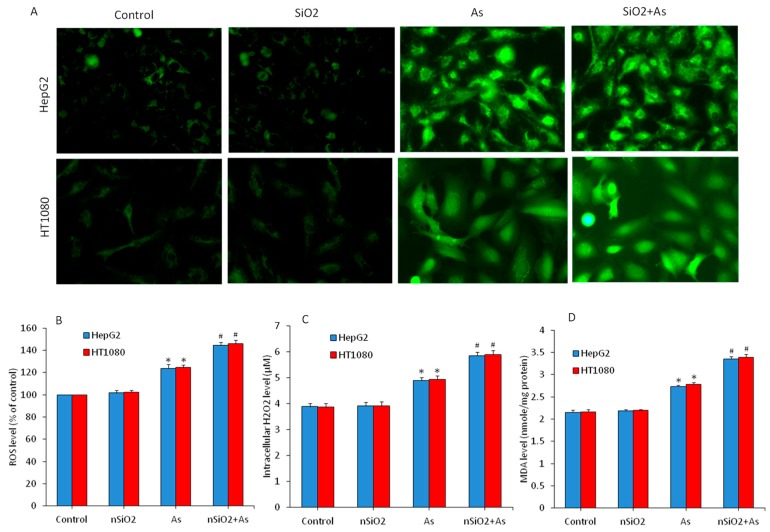
Pro-oxidant levels in HepG2 and HT1080 cells exposed for 24 h to nSiO_2_ (10 µg/mL), As (1 µg/mL) or SiO_2_ + As (10 µg/mL + 1 µg/mL) for 24 h. (**A**) Fluorescent microscopic images of intracellular reactive oxygen species (ROS) level. (**B**) Quantitative level of intracellular ROS level. (**C**) Intracellular H_2_O_2_ level. (**D**) Malondialdehyde (MDA) level. Data are presented as the mean ± SD of three independent experiments (n = 3). * indicates significant effect in comparison to the control group (*p* < 0.05). ^#^ indicates significant effect in comparison to the nSiO_2_ group alone or As group alone (*p* < 0.05).

Oxidative stress is one of the potential mechanisms through which nanoparticles exert toxicity to human cells [45]. Heavy metals and metalloids also have potential to induce toxicity through the disturbance of redox homeostasis [46]. Hence, we further evaluated the combined effects of nSiO_2_ and As on several parameters of oxidative stress in HepG2 and HT1080 cells exposed to nSiO_2_ and/or As for 24 h. The ROS, H_2_O_2_ and MDA were assayed as pro-oxidant markers. Microscopic data have shown that ROS level (DCF fluorescence) in nSiO_2_ was not different from the control group (Figure 3A). However, DCF fluorescence intensity (ROS level) was increased in the As group compared to those of the control group. Interestingly, DCF fluorescence intensity in the co-exposure group (nSiO_2_ + As) was more pronounced than those of the As group alone (Figure 3A). In agreement with microscopy data, quantitative data have also shown that ROS level in the nSiO_2_ group was similar to the control group, but was significantly higher in As group in comparison to the control group (Figure 3B) (*p* < 0.05). Interestingly, ROS level was significantly increased in the co-exposure group (nSiO_2_ + As) in comparison to the As group alone (Figure 3B) (*p* < 0.05). Combined effects of nSiO_2_ and As were further examined through H_2_O_2_ and MDA levels. Figure 3C,D demonstrated that H_2_O_2_ and MDA levels in nSiO_2_ group were not different from the control group, but significantly higher in the As group. Again, H_2_O_2_ and MDA levels in the co-exposure group (nSiO_2_ + As) were significantly higher in comparison to the As group alone (*p* < 0.05). These data suggested that co-exposure of nSiO_2_ and As exacerbated the oxidative damage of HepG2 and HT1080 cells than those of the As exposure alone.

**Figure 4:**
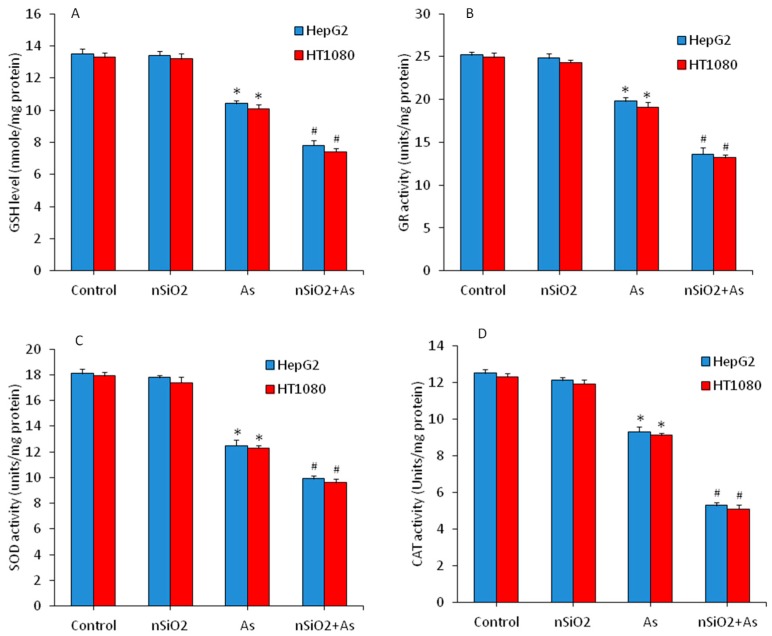
Antioxidant levels in HepG2 and HT1080 cells after exposure to nSiO_2_ (10 µg/mL), As (1 µg/mL) or SiO_2_ + As (10 µg/mL + 1 µg/mL) for 24 h. (**A**) Intracellular glutathione (GSH) level. (**B**) Glutathione reductase (GR) enzyme activity. (**C**) Superoxide dismutase (SOD) enzyme activity. (**D**) Catalase (CAT) enzyme activity. Data are presented as the mean ± SD of three independent experiments (n = 3). * indicates significant effect in comparison to the control group (*p* < 0.05). ^#^ indicates significant effect in comparison to the nSiO_2_ group alone or As group alone (*p* < 0.05).

Equilibrium between pro-oxidants generation and their elimination by antioxidants is a very delicate phenomenon. Excessive generation of pro-oxidants or depletion of antioxidants may cause oxidative damage of cellular components [47]. Antioxidant molecules and enzymes play a crucial role in scavenging the free oxygen radicals [48]. SOD enzyme dismutates the highly reactive superoxide anion (O_2_) into comparatively less reactive H_2_O_2_. GSH, GR and CAT further reduce the H_2_O_2_ into water (H_2_O) and molecular oxygen (O_2_) by various mechanisms [49]. In the present study, we assessed the effects of nSiO_2_ and/or As on antioxidants in HepG2 and HT1080 cells. As we can see in Figure 4A–D antioxidant molecule GSH and antioxidant enzymes (GR, SOD and CAT) activity were lower in the As group in comparison to the control group (*p* < 0.05). Interestingly, cells co-exposed to nSiO_2_ and As showed significantly higher reduction in antioxidant levels than cells exposed to As alone (Figure 4) (*p* < 0.05). Overall, these results showed increased ROS, H_2_O_2_ and MDA along with decreased GSH, GR, SOD and CAT, suggesting higher oxidative stress after combined exposure of nSiO_2_ and As in comparison to As group alone.

There are increasing evidences that nSiO_2_ might enhance the oxidative stress mediated toxicity of other chemicals or environmental pollutants. Guo et al. [42] observed that nSiO_2_ significantly enhances Cd-induced oxidative damage in mice liver. Yu et al. [43] observe synergistic toxicity of nSiO_2_ and MeHg on oxidative stress markers (ROS generation, lipid peroxidation and depletion of antioxidants) in A549 cells. Co-exposure of 70 nm nSiO_2_ (SP70) to mice potentiate the oxidative stress mediated hepatotoxicity of acetaminophen and tetracycline [50]. Combined exposure of nSiO_2_ and benzo(a)pyrene enhance the MDA content and reduce the SOD and glutathione peroxidase in human BEAS-2B cells [23].

### 3.4. Apoptosis Study

**Figure 5:**
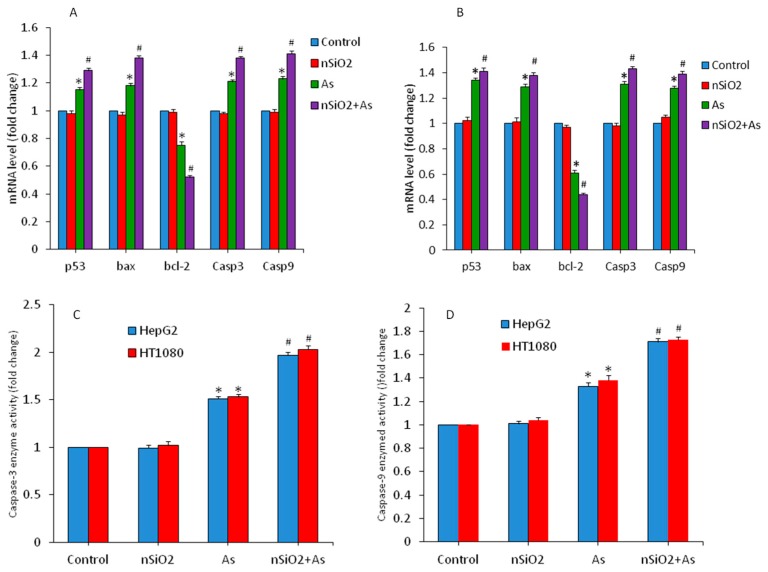
Expression of apoptotic genes and enzymes in HepG2 and HT1080 cells exposed for 24 h to nSiO_2_ (10 µg/mL), As (1 µg/mL) or SiO_2_ + As (10 µg/mL + 1 µg/mL). (**A**) mRNA level of apoptotic genes in HepG2 cells. (**B**) mRNA level of apoptotic genes in HT1080 cells. (**C**) Activity of caspase-3 enzymes. (**D**) Activity of caspase-9 enzymes. Data are presented as the mean ± SD of three independent experiments (n = 3). * indicates significant effect in comparison to the control group (*p* < 0.05). ^#^ indicates significant effect in comparison to the nSiO_2_ group alone or the As group alone (*p* < 0.05).

Apoptosis (programmed cell death) is regulated by a number of genes [51]. Free oxygen radicals such as the superoxide anion (O_2̇_˙) serves as signaling molecules in the apoptotic process [52]. Antioxidant GSH has been linked with a large panel of actions controlling gene expression and apoptotic pathways [53]. Studies have shown that nSiO_2_ is able to induce mitochondria mediated apoptosis [6,9,54]. As is also known for apoptosis induction through free oxygen radical generation [55,56]. Hence, we examined the effects of nSiO_2_ and/or As on the regulation of several apoptotic genes (*p53*, *bax*, *bcl-2*, *casp3* and *casp9*) in HepG2 and HT1080 cells. We observed that that nSiO_2_ did not change the regulation of these apoptotic genes. However, As exposure increased the mRNA expression of *p53* (tumor suppresser) and *bax* (pro-apoptotic) genes, while decreased the expression of the *bcl-2* gene (anti-apoptotic) in comparison to the control group (Figure 5A,B) (*p* < 0.05). Moreover, apoptotic genes *caspase-3* and *caspase-9* were also up-regulated in As treated cells. Interestingly, apoptotic responses were more pronounced in the co-exposure group (SiO_2_ + As) than those of As group alone.

Activity of caspase-3 and caspase-9 enzymes was further examined to confirm the mRNA results. We found that activity of these apoptotic enzymes were significantly higher in the As group in comparison to the control group. Again, in co-exposure group activity of caspase-3 and caspase-9 enzymes were significantly higher than those in the As group alone. (Figure 5C,D). Hence, non-cytotoxic concentration of nSiO_2_ increased the severity of As on the altered regulation of apoptotic genes. Yang et al. [24] demonstrated that up-regulation of pro-apoptotic proteins e.g., bax, caspase-3 and caspase-9 and down-regulation of anti-apoptotic protein bcl-2 due to MeHg were aggravated by nSiO_2_ exposure in cardiac cells and tissues.

**Figure 6:**
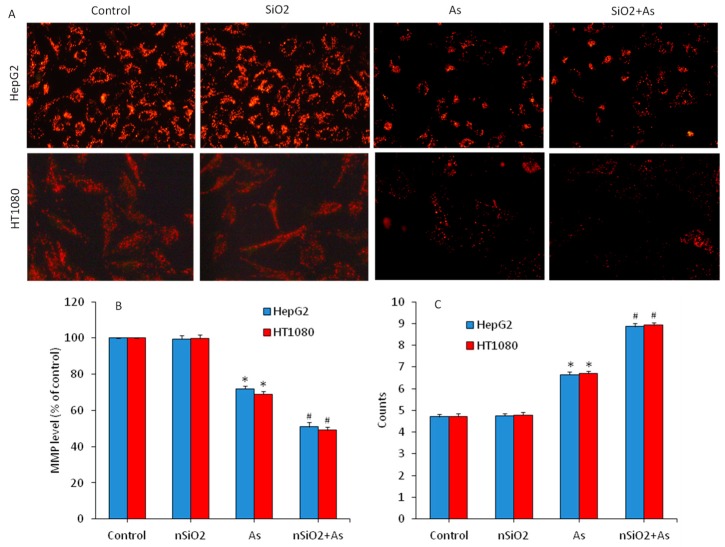
Mitochondrial membrane potential (MMP) level and cell cycle phases of HepG2 and HT1080 cells exposed for 24 h to nSiO_2_ (10 µg/mL), As (1 µg/mL) or SiO_2_ + As (10 µg/mL + 1 µg/mL). (**A**) Fluorescent microscopic images of MMP level (rhodamine-123 (Rh-123) probe). (**B**) Quantitative level of MMP. (**C**) SubG1 phases of cell cycle. Data are presented as the mean ± SD of three independent experiments (n = 3). * indicates significant effect in comparison to the control group (*p* < 0.05). ^#^ indicates significant effect in comparison to the nSiO_2_ group alone or the As group alone (*p* < 0.05).

Decreased mitochondrial membrane potential (MMP) is also linked with apoptotic cell death [54,57]. We further evaluated the MMP level in HepG2 and HT1080 cells exposed for 24 to nSiO_2_ and/ or As. Figure 6A showed that fluorescent intensity of Rh-123 (indicator of MMP level) in the nSiO_2_ group was almost similar to the control group. However, the MMP level was significantly decreased due to As exposure (*p* < 0.05). Moreover, MMP depletion in the co-exposure group (nSiO_2_ + As) was more pronounced as compared to the As group alone (*p* < 0.05). Similar to microscopy data, quantitative analysis also demonstrated that MMP level in the nSiO_2_ group was not different from the control group (Figure 6B) but significantly decreased in the As group. Again, MMP loss in the co-exposure group (nSiO_2_ + As) was more pronounced than those of the As group alone (*p* < 0.05) (Figure 6B). A recent study shows that the non-cytotoxic concentration of nSiO_2_ significantly aggravates Pb-induced up-regulation of bax, caspase-3 and caspase-9 proteins, and down-regulation of bcl-2 protein along with MMP loss in human lung (A549) cells [27].

At the end we explored the effects of nSiO_2_ and/or As exposure on cell cycle phases of HepG2 and HT1080 cells. It is known that cells with damaged DNA accumulate in G1 (gap1), S (DNA synthesis), or G2/M (gap2/mitosis) phases. However, cells with irreversibly damaged DNA get eliminated through programmed cell death (apoptosis) by accumulating in the subG1 phase. Our flow cytometer analysis showed that cell cycle progression in the nSiO_2_ group was similar to the control group. However, As-induced apoptosis in both HepG2 and HT1080 cells (Figure 6C). In As group, cell gathering in SubG1 phase was significantly higher (6.65% of HepG2 and 6.71% of HT1080) in comparison the control group (4.71% of GepG2 and 4.73% of HT1080) (*p* < 0.05). Intriguingly, due to combined exposure of nSiO_2_ and As the gathering of cells in SubG1 phase was significantly higher (8.86% of HepG2 and 8.93% of HT1080) than those of the As group alone (Figure 6C) (*p* < 0.05). Altogether, these results demonstrated that non-cytotoxic concentration of nSiO_2_ exposure alone did not provoke apoptosis in both HepG2 and HT1080 cells, but effectively exacerbated mitochondrial mediated apoptosis when co-exposed with As.

## 4. Conclusions

We explored the combined effects of nSiO_2_ and As in human liver cells (HepG2) and human fibroblasts (HT1080). Results demonstrated that nSiO_2_ were not toxic, however, As significantly caused toxicity to both types of cells. Interestingly, non-cytotoxic concentration of nSiO_2_ significantly augmented the toxic effects of As. Co-exposure of nSiO_2_ and As caused cell viability reduction, generation of pro-oxidants (ROS, H_2_O_2_ and MDA) and depletion of antioxidants (GSH, GR, SOD and CAT). Combined exposure of nSiO_2_ and As induced apoptosis through changing the regulation of apoptotic genes and cell cycle phases along with MMP depletion. Augmentation of As-induced toxicity by nSiO_2_ may be due to specific properties of nSiO_2_ as a carrier, which facilitated cellular entry of As. This study warrants future work to explore the co-exposure effects of nSiO_2_ and As in a suitable animal model in order to provide more insights into molecular mechanisms involved in their combined toxicity.

## Figures and Tables

**Table 1:** Hydrodynamic size and zeta potential measurement of nSiO_2_ in distilled water and culture medium.

Time	Distilled Water		DMEM	
	Hydrodynamic Size (nm)	Zeta Potential (mV)	Hydrodynamic Size (nm)	Zeta Potential (mV)
0 h	88.31 ± 2.22	−32.56 ± 1.12	93.25 ± 1.56	−33.23 ± 0.95
4 h	87.52 ± 1.87	−31.88 ± 0.98	92.34 ± 1.11	−33.42 ± 0.83
8 h	89.16 ± 2.17	−32.36 ± 1.32	91.78 ± 1.48	−32.87 ± 0.86
24 h	89.84 ± 2.56	−31.11 ± 1.25	93.59 ± 1.73	−33.17 ± 0.75

DMEM: Dulbecco’s modified Eagle’s medium.
